# Supplementary material for: Ambient-pressure 151-K superconductivity in HgBa2Ca2Cu3O8+δ via pressure quench
Source: Proc Natl Acad Sci U S A. 2026 Mar 9;123(11):e2536178123. doi: 10.1073/pnas.2536178123 (PMC12993975; doi:10.1073/pnas.2536178123)
Supplement: Supplementary file 1 — Appendix 01 (PDF) [file pnas.2536178123.sapp.pdf]

PNAS

Supporting Information for
Ambient-pressure 151-K superconductivity in $HgBa_2Ca_2Cu_3O_{8+\delta}$ *via* pressure quench

Liangzi Deng[1,*], Thacien Habamahoro[1], Artin Safezoddeh[1], Bishnu Karki[1], Sudaice Kazibwe[1], Daniel J. Schulze[1], Zheng Wu[1], Matthew Julian[2], Rohit P. Prasankumar[2], Hua Zhou[3], Jesse S. Smith[3], Pavan R. Hosur[1], Ching-Wu Chu[1,*]

Liangzi Deng, Ching-Wu Chu
Email: cwchu@uh.edu, ldeng2@central.uh.edu

This PDF file includes:

Figures S1 to S8

Figures

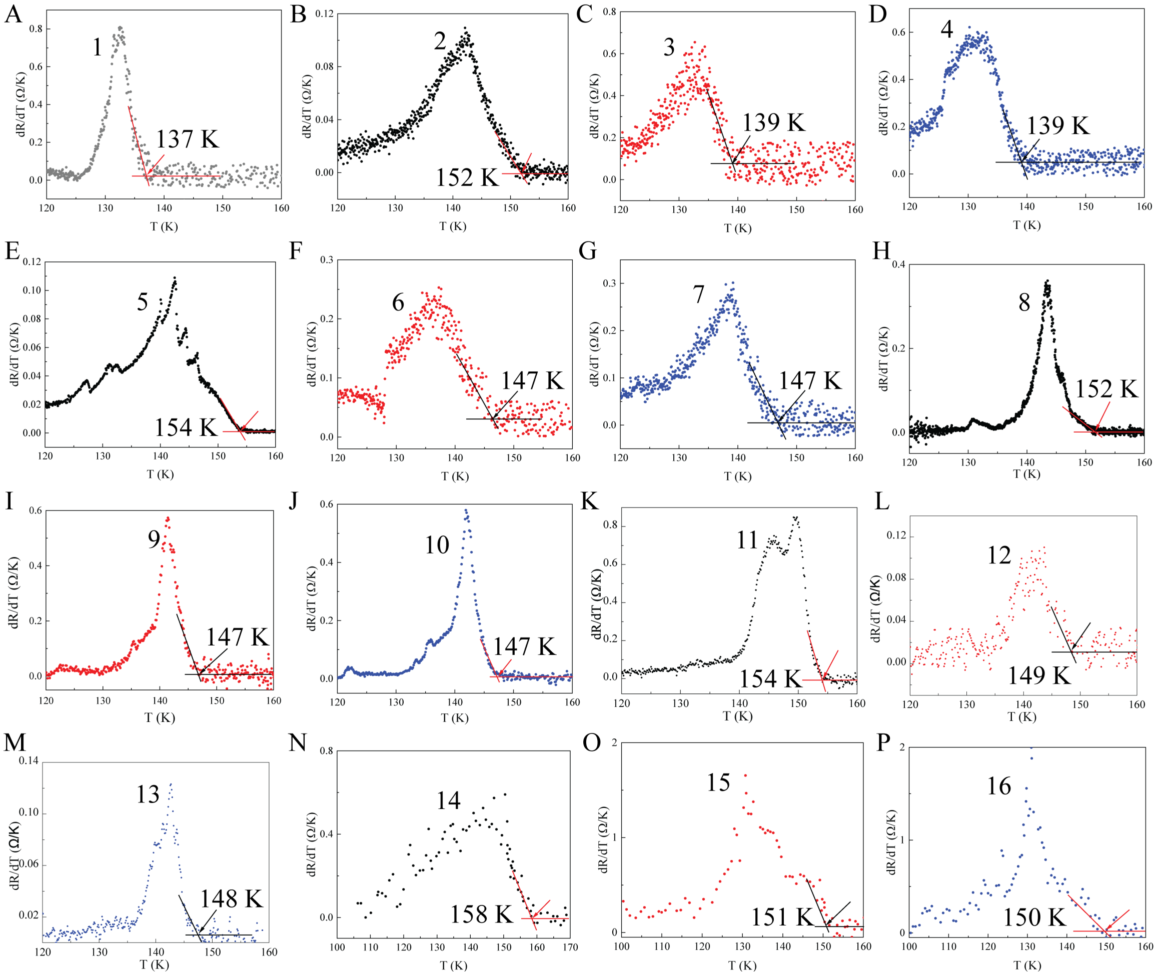

Fig. S1. (A–P) Temperature-dependent dR/dT results for R(T) curves 1–16, respectively, in Fig. 2, which are used for determining the corresponding onset T_cs.

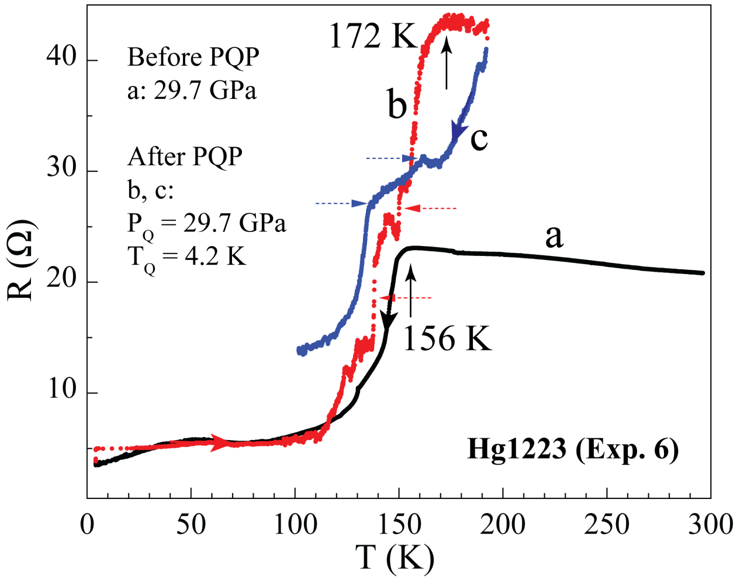

Fig. S2. Temperature-dependent resistance R(T) for Hg1223 (S5) under different conditions before and after PQP. a—before PQP under 29.7 GPa on cooling; and after PQP at P_Q = 29.7 GPa and T_Q = 4.2 K, b—on warming and c—on cooling. Dashed arrows indicate possible superconducting transitions retained after PQP during different thermal cycles.

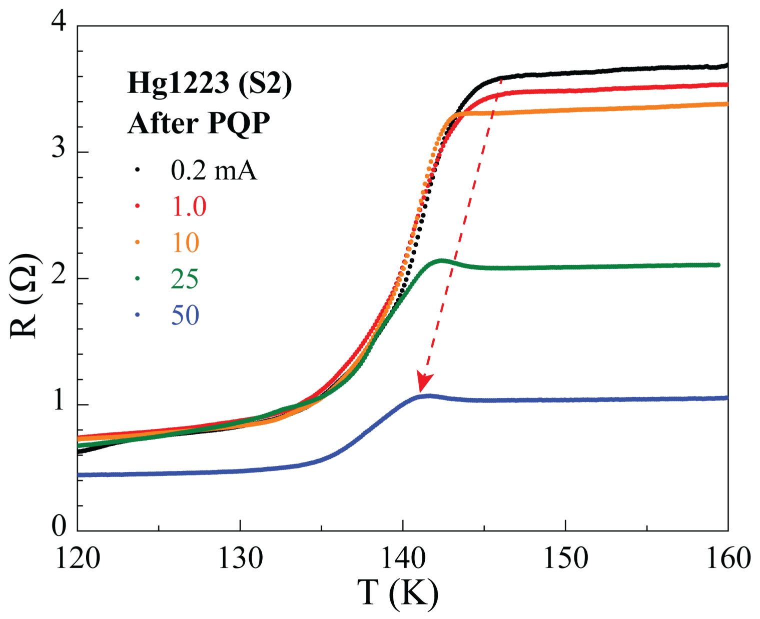

Fig. S3. Parallel downward shifting of R(T) with increasing current confirms the superconducting nature of the transition for Hg1223 sample S2.

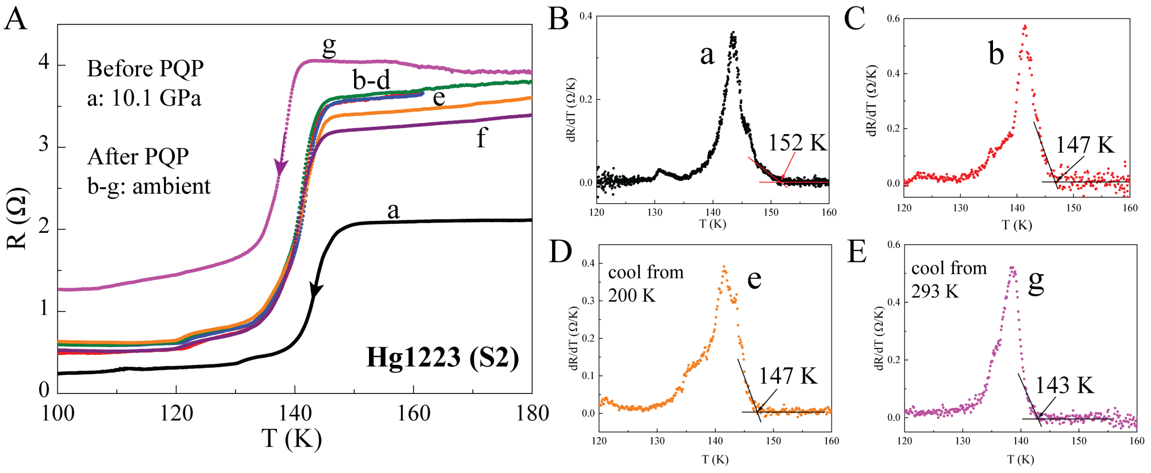

Fig. S4. Thermal stability testing of Hg1223 (S2). (A) a—before PQP under 10.1 GPa on cooling; and after PQP at P_Q = 10.1 GPa and T_Q = 4.2 K, b—on warming, c—on cooling from 160 K, d—on warming, e—on cooling from 200 K, f—on warming, and g—on cooling from 293 K (R/3). Panels at right (B–E): corresponding dR/dT *vs.* T data to determine the T_cs for curves a, b, e, and g, respectively.

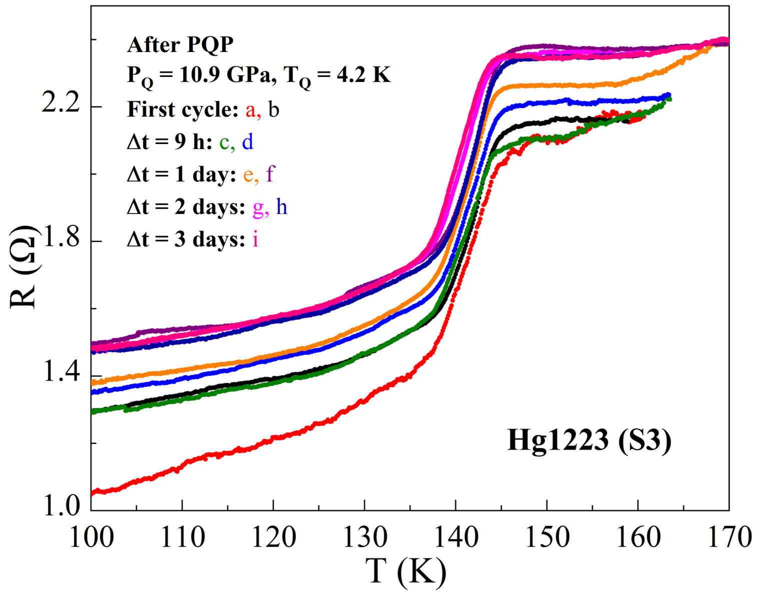

Fig. S5. Temporal stability testing of Hg1223 (S3). Immediately after PQP at P_Q = 10.9 GPa and T_Q = 4.2 K, a—on warming to 160 K and b—on cooling; 9 hours after PQP, c—on warming to 160 K and d—on cooling; 1 day after PQP, e—on warming to 170 K and f—on cooling; 2 days after PQP, g—on warming to 160 K and h—on cooling; 3 days after PQP, i—on warming to 170 K.

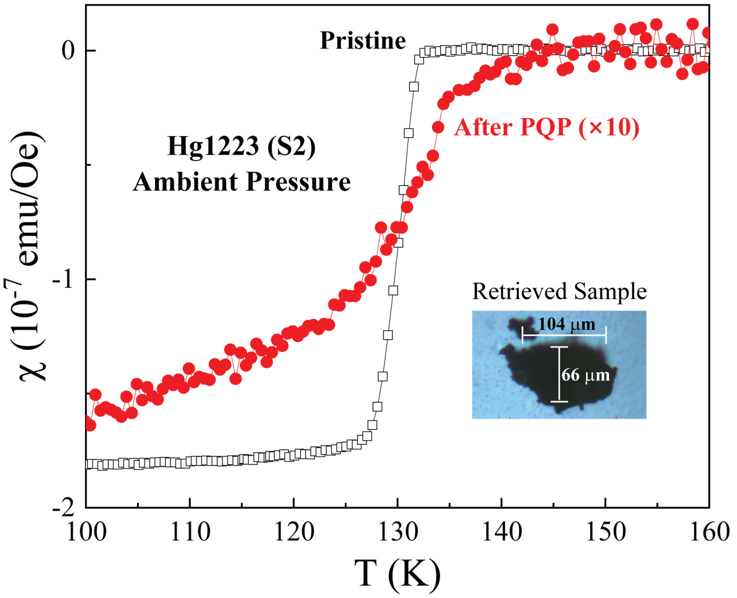

Fig. S6. DC magnetic results for pristine Hg1223 crystal #1 (source of S2) before PQP (black squares) and for Hg1223 sample S2 following retrieval from the DAC after PQP (red circles). Inset: part of Hg1223 (S2) retrieved from the DAC after PQP.

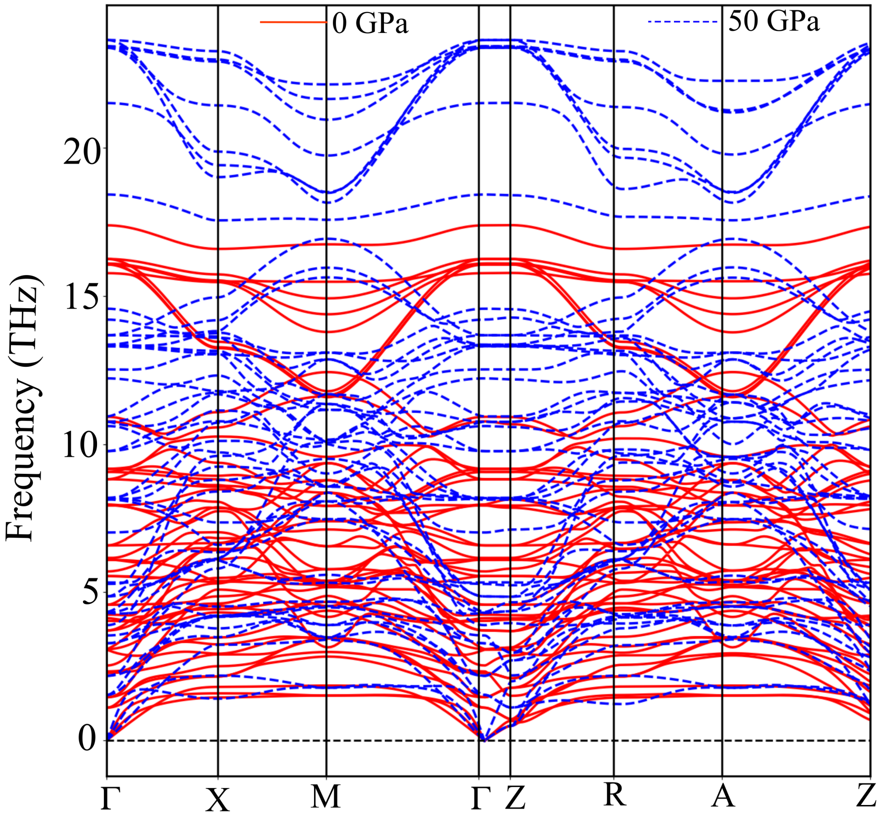

Fig. S7. Comparison of phonon dispersion in Hg1223 between 0 and 50 GPa. The absence of imaginary modes indicates structural stability without any structural transition up to 50 GPa.

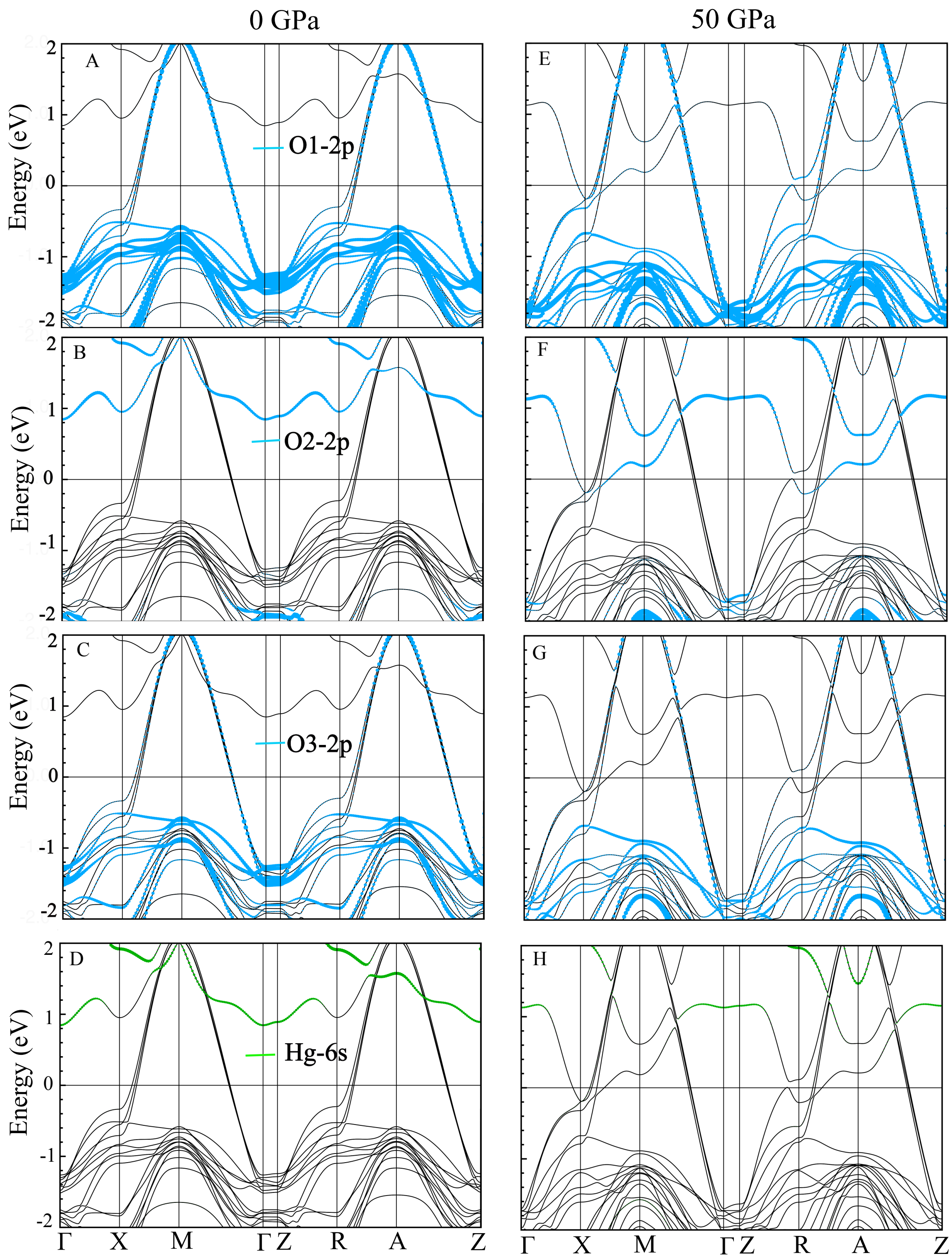

Fig. S8. Projection of orbital contributions on DFT bands from different oxygen sites (O1 and O3 from the Cu-O planes and O2 being the apical oxygen) and from Hg under ambient pressure and under high pressure. (A and E) O1, (B and F) O2, (C and G) O3, and (D and H) Hg for 0 GPa and 50 GPa, respectively. By comparing (B) and (F), it is clear that the O2-2p orbitals contribute at the Fermi level under high pressure.
